# Maintaining Cognitive Performance at the Expense of Gait Speed for Asymptomatic Concussed Athletes: A Novel Dual-Task and Post-Exercise Assessment

**DOI:** 10.3390/brainsci14070715

**Published:** 2024-07-17

**Authors:** Gabriel Lavoie, Mathieu Bolduc, Veronik Sicard, Franco Lepore, Dave Ellemberg

**Affiliations:** 1 Psychology, University of Montreal, Montréal, QC H3T 1J4, Canada; lavoiegabriel1234@hotmail.com (G.L.); mathieu.bolduc.2@umontreal.ca (M.B.); franco.lepore@umontreal.ca (F.L.); 2 CHEO Research Institute, Ottawa, ON K1H 5B2, Canada; veronik.sicard@gmail.com; 3 Kinesiology, University of Montreal, Montréal, QC H3T 1J4, Canada

**Keywords:** mild traumatic brain injury, cognition, gait, physical exercise, dual-task

## Abstract

Our goal was to evaluate persisting deficits in gait and executive functioning in asymptomatic athletes with a history of concussion using a novel approach combining a dual-task paradigm and post-exercise exertion. Thirty-eight athletes aged 17 to 25 years old participated in the study, including 18 with a history of concussion. The dual-task paradigm required walking continuously at a predetermined self-paced target speed of 6.5 km/h while executing a complex switch task. Athletes completed two conditions, each on separate days: (1) dual task alone and (2) dual task following 20 min of running on a non-motorized treadmill. The statistical analyses revealed a significant reduction in gait speed exclusively for athletes with a history of concussion and only following the post-exercise condition (*p* = 0.008). These findings suggest that although asymptomatic concussed athletes maintain a cognitive performance comparable to non-concussed athletes, this appears to be achieved at the expense of gait speed. Our results underscore the importance of incorporating gait assessments and post-exercise exertion into concussion evaluation protocols in both research and clinical settings.

## 1. Introduction

Over the past two decades, it has become widely recognized that sport-related concussions (SRCs) constitute a significant public health concern [1]. Research indicates that the incidence of SRCs in contact sports is notably high, at approximately 4.18 per 1000 athlete exposures, contributing to a staggering 450,000 hospital emergency visits related to concussions each year in the U.S. [2,3]. Inadequately managed SRCs significantly increase the risk of poor health outcomes, including a higher probability of reinjury and persistent, long-term effects [4]. A major challenge in effectively addressing SRCs is the reliance on self-reported symptoms for assessment and management, which can lead to underreporting and mismanagement [5].

Indeed, athletes can deliberately misreport their symptoms to mask their injury or to expedite their return to sports [5,6]. Furthermore, athletes who no longer report symptoms can continue showing significant cognitive impairments beyond the initial injury [7,8,9,10]. Advanced imaging technologies and electroencephalography have revealed persistent alterations affecting asymptomatic athletes [11,12,13,14,15]. This body of literature suggests that the absence of symptoms does not necessarily mean an athlete has fully recovered.

To improve SRC detection and management, cognitive assessments have become increasingly important. However, these assessments are typically conducted under resting conditions, which do not reflect the dynamic environment of their sports activities, in which athletes must perform cognitively demanding tasks while in constant motion. Numerous studies have shown that physical activity can impact the cognitive functioning of concussed athletes. For instance, two studies employing a repeated assessment of cognitive performance using the ImPACT test battery during the acute phase of injury (when athletes are ready to return to sport) found that 28% of athletes exhibited lower cognitive performance after engaging in moderate aerobic exercise [16,17]. Another study by our group highlighted the added value of post-exercise assessments in identifying ongoing alterations that may have been unnoticed at rest [7]. The study employed a complex task that tests executive functions (known as the switch task) and found that 30% of athletes with concussions exhibited impaired cognition, with an additional 10% of athletes demonstrating impairments only detectable after engaging in 20 min of moderate-to-vigorous exercise. A follow-up study found that 21% of athletes who sustained a concussion at least 6 months prior to testing showed cognitive impairments after exercise, compared to 3% before the exercise [18]. Overall, these studies support the suggestion that clinical evaluations of concussions should incorporate a physical exertion protocol to enhance task sensitivity in detecting persisting impairments following a concussion. This was also highlighted in the last concussion consensus [19].

Most sports require athletes to simultaneously engage in motor and cognitive processes, such as running while rapidly planning strategies and making decisions. The allocation of resources to both sets of processes can increase the burden on athletes, elevating the risk of symptom resurgence and the potential for new injuries. Protocols assessing both motor and cognitive functions are known as dual-task paradigms. Performance declines in dual tasks are attributable to the competition for limited cognitive resources [20]. Research suggests that dual-task assessments are more effective than cognitive single tasks for concussed athletes [21,22,23]. The complexity of these dual tasks is a critical factor in sensitivity, with more complex tasks demonstrating a greater ability to detect impairments following a concussion [22,24]. Recognizing the efficacy of these assessments, leading sports medicine authorities have revised their guidelines to incorporate dual tasks. For example, the latest consensus from the Concussion In Sport Group (CISG) considers dual-task assessments an integral part of the comprehensive concussion assessment [19]. Therefore, including a dual-task paradigm in the cognitive evaluation of concussed athletes is essential for a more objective decision-making process regarding the return-to-play timeline.

To the best of our knowledge, no prior study has integrated dual-task protocols into post-exercise assessments, representing the next logical progression for improving the sensitivity of post-concussion evaluations.

Accordingly, the objective of this study was to integrate an experimental dual-task paradigm that mirrors the real-world challenges athletes face during competitive sports with a physical exertion protocol. Our dual task required participants to engage in higher-level cognitive processing, which required rapid decision-making, while continuously monitoring their gait. The complexity of the gait task was increased by instructing participants to maintain a gait speed slightly faster than their normal walking pace. The cognitive task employed a complex color–shape visual switch task developed and validated by our lab [7,18,25,26]. Dual-task performance was assessed in a group of asymptomatic athletes with a history of concussion and compared with that of athletes with no history of concussion. The exercise protocol consisted of 20 min of moderate aerobic running on a non-motorized treadmill. Athletes performed the dual task during two sessions: once after a rest period and once after the physical exertion protocol.

Given that similar exercise protocols can lead to an increase in cognitive performance in non-concussed individuals [27,28,29,30], we expected that the control group would show improved dual-task performance (a faster reaction time, better response accuracy, and steadier gait) following exercise. We hypothesized that the concussion group would not only underperform compared to the control group on the dual task, but also demonstrate worse performance when the dual task was performed post-exercise.

## 2. Materials and Methods

### 2.1. Participants

This study was conducted with the approval of the Clinical Research Ethics Committee of the Université de Montréal, adhering to the ethical standards outlined in the 1964 Declaration of Helsinki. Informed consent was obtained from all participants before their inclusion in the study.

Various colleges and universities in the Montreal area were contacted to recruit participants by reaching out to athletic directors and team coaches. Additionally, participants referred their teammates to the study when appropriate.

Participants were excluded if they had any known psychiatric, neurological, or neurodevelopmental disorders; a history of head trauma beyond sports-related concussions (or all head trauma for the control group); a current use of psychoactive medications; color blindness; a history of using illicit substances (e.g., stimulants, opiates, marijuana, sedatives) or consuming more than ten alcoholic beverages per week; scored higher than the set thresholds on the Beck Anxiety Inventory (BAI-16; Pearson assessment, Canada), Beck Depression Inventory-II (BDI-II-19; Pearson assessment, Canada) or Wender-Utah Rating Scale (WURS-48; Utah, USA).

All participants in the concussion group had a history of at least one medically diagnosed sport-related concussion. They had successfully undergone a complete return to both sport and school activities from their last concussion and did not report any symptoms of concussion at the time of testing.

### 2.2. Questionnaires and Instruments

#### 2.2.1. Questionnaires

A comprehensive general information questionnaire was used to conduct a structured interview to acquire data pertaining to the inclusion and exclusion criteria, as well as demographic and concussion-related information such as age, sex, years of education, and time since concussion.

Moreover, all participants were asked: “Following a blow to the head, neck, or body, have you ever experienced any concussion-like symptoms?” Subsequently, they were presented with the list of clinical symptoms from the Sports Concussion Assessment Tool (SCAT-5) [31]. Any participant from the control group responding “yes” to this question was excluded from participating in the study.

Participants completed three self-reported questionnaires to screen for depression, anxiety, and ADHD. The Beck Anxiety Inventory (BAI) is a 21-item self-report questionnaire that measures the severity of common anxiety symptoms over the past week, with a scoring range of 0–63; higher scores indicate more severe anxiety symptoms. The Beck Depression Inventory (BDI) is a 21-item self-report questionnaire that measures the severity of depression symptoms over the past two weeks, with a scoring range of 0–63; higher scores indicate more severe depression symptoms. The Wender-Utah Rating Scale (WURS) is a 25-item self-report questionnaire that retrospectively assesses the presence of ADHD symptoms during childhood, with a scoring range of 0–100; higher scores indicate more ADHD symptoms. Participants with scores exceeding the set thresholds—16 for the BAI, 19 for the BDI, and 48 for the WURS—were not invited to participate in the study.

#### 2.2.2. Curve Trainer Woodway Treadmill

A non-motorized treadmill was utilized (Curve Trainer Woodway Treadmill, USA-manufactured, Waukesha, WI, USA), as it allows participants to control their speed. This treadmill was selected for this feature, as gait speed can serve as a reliable outcome measure. The Woodway CurvePro 1.5 software provides 75 speed data points per second, ensuring a high level of precision for gait speed.

#### 2.2.3. Polar H10 Heart Rate Monitor

The Polar H10 heart rate monitor (Polar, Kempele, Finland) chest strap was used to monitor the cardiac frequency of each participant throughout the exercise protocol. The device is worn around the chest, where it measures heart rate using electrical signals with a sampling frequency of 1000 Hz.

#### 2.2.4. Switch Task—Color–Shape Version

**Figure 1:**
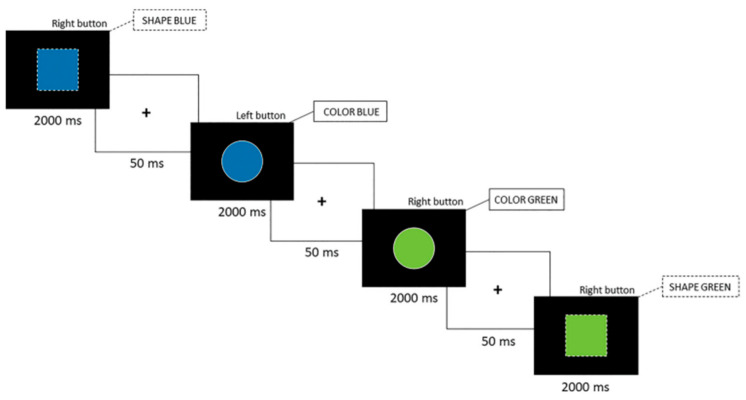
Color–shape switch task.

The color–shape switch task used in this study was adapted from our lab’s previous research [25,26]. The task consisted of three distinct conditions, each involving a different set of rules, each presented in a different condition. The two initial conditions are homogeneous, requiring participants to respond based on either color (green or blue) or shape (square or circle). The third condition introduces heterogeneity by combining both rule sets. During this condition, participants’ responses are cued by the stimulus’s outline (solid = color rule; dashed = shape rule). Figure 1 provides a visual representation of the task.

The stimuli were presented for a maximum duration of 2000 ms, with a 50 ms delay between the participants’ response and the next stimulus. Each of the two homogeneous conditions included 60 items and lasted approximately 126 s. The heterogeneous conditions consisted of two distinct blocks, each containing 120 items and lasting approximately 250 s.

An alternative version of the task was used to reduce the practice effects associated with repeated testing. This version closely resembled the original task, with a key modification: the rule sets were reversed (solid line = shape; dashed line = color). Our research suggests that this alternative version successfully reduces the practice effect [25,26].

Figure 1 depicts the color–shape switch task used in the current study. The call-out states the rule set used to elicit the appropriate response. For example, the first stimulus shows a blue square with a dashed outline. Participants have to respond according to the shape, hence the right button.

### 2.3. Description of the Dual Task

Participants concurrently performed the switch task (comprising the two homogeneous phases and the heterogeneous phases) and the gait task (maintaining a gait speed as close as possible to 6.5 km/h).

### 2.4. Exercise Protocol

The target heart rate zone was set at 80–90% of the age-predicted maximum heart rate [32]:

HRmax = 208 − (0.7 × age)(1)

The exercise task involved 20 min of running on a non-motorized treadmill, starting when the participant’s heart rate reached the lower end of this target range (80%). Throughout the exercise protocol, participants were instructed to maintain a consistent pace throughout the exercise. If their heart rate dropped below 80%, they were prompted to increase their speed; if it exceeded 90%, they were advised to decelerate. This protocol is based on accumulated findings indicating that 20 min of moderate aerobic exercise can enhance cognition [27,28,29,30,33,34,35]. The participant did not have access to their heart rate, but relied on the administrator’s instructions.

### 2.5. Procedure

The study was conducted over two sessions, on different days. The dual-task condition was administered during the first session, whilst the post-exercise dual-task condition was administered during the second session. Participants were asked to be well rested before each session and not to engage in any form of physical exertion.

#### 2.5.1. Session 1: Dual-Task Condition

The first session of the study began with obtaining informed consent, conducting a structured interview, and administering screening questionnaires. Participants were then fitted with the Polar H10 chest strap. They were familiarized with the non-motorized treadmill by practicing accelerating and decelerating their walking speed. Once comfortable, participants were tasked with maintaining a target walking speed of 6.5 km/h during a three-minute gait practice session. Subsequently, they performed the dual task, utilizing the indicator panel to adjust their speed as needed, so they had continuous access to their speed. This setup allowed them to maintain their speed while allocating attentional resources to the gait task due to direct feedback. The session lasted approximately 1 h and 15 min, including a 10 min rest period at the beginning and end of the session.

#### 2.5.2. Session 2: Post-Exercise Dual-Task Condition

The session began with participants being fitted with the Polar H10 chest strap, followed by a 10 min rest period. They then engaged in the 20 min exercise protocol on the treadmill. After exercise, a brief cooldown period was allowed before completing the dual task. The session ended with another 10 min rest period and lasted approximately 1 h and 15 min.

### 2.6. Study Design

This study is a quantitative quasi-experimental cross-sectional study that recruited volunteer athletes and assigned them to either the control or concussion group based on their adherence to our inclusion criteria. Thus, no random assignment was made between groups.

### 2.7. Statistical Analyses

Demographic information was analyzed using a series of *t*-tests and chi-square tests with SPSS 28.0 for Windows (IBM, Armonk, NY, USA).

For the cognitive outcome variables, the average reaction time and accuracy were calculated only for the heterogeneous condition, which is the component of the task that taps into executive functioning [25]. The gait outcome variable (gait speed) was obtained by computing the average speed (km/h) that each participant maintained during the heterogeneous phase of the switch task.

Outcome variables were analyzed using repeated measures 2 × 2 (Group [Concussion, Control] × Condition [No-Exercise, Post-Exercise]) ANOVAs. Post hoc analyses were conducted using paired *t*-tests with Bonferroni corrections to verify the differences between the two conditions within each group, and independent *t*-tests were used to examine group differences at each condition, even in the absence of an interaction as an exploratory analysis.

A power analysis conducted with G*Power (version 3.1.9.7) suggested that, to detect an interaction using a repeated measures ANOVA (two-tails, f = 0.25, α ˂ 0.05, power 0.8), a total sample size of 34 participants is required. Moreover, to detect a bivariate correlation (two-tails, r = 0.3, α ˂ 0.05, power 0.8) the sample size should be 84 participants.

## 3. Results

### 3.1. Participant Demographics

Eighteen athletes had experienced at least one medically diagnosed concussion (average of 1.83 ± 0.22) within the range of 3.45 to 10.49 months (average of 6.01 ± 1.92) prior to testing (Concussion group), while the other twenty were never diagnosed with a concussion nor did they self-report any history of concussion (Control group). The mean time to symptom recovery for concussed athletes was 20.71 ± 9.81 days. They all achieved full recovery within the first three weeks, except for two individuals who took 4 months to recover. They suffered an average of 12.10 ± 1.23 symptoms and reported a mean intensity of 33.06 ± 4.72. The concussed athletes also reported an average of 1.17 ± 0.60 suspected concussions that were not medically diagnosed. Athletes from either group did not report any symptoms before, during, or after completing our protocol in both sessions.

Demographic information is presented in Table 1. Independent *t*-tests and chi-square tests did not reveal any significant group differences for their BDI, BAI, Wender-Utah, sex, years of education, and age.

### 3.2. Cognitive Outcomes

#### 3.2.1. Switch Accuracy

**Figure 2:**
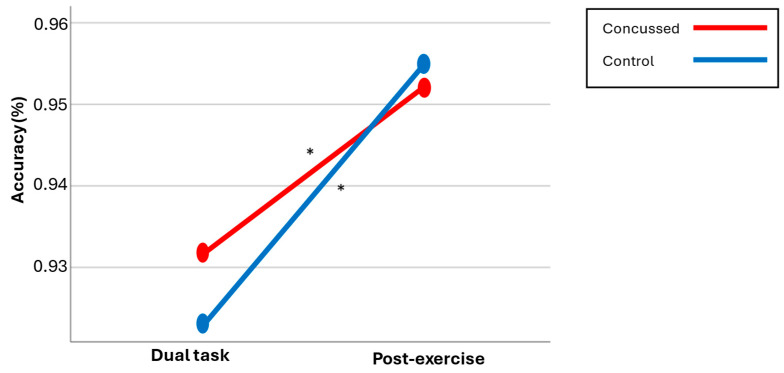
Cognitive accuracy across both conditions.

No significant Group × Condition interaction, *p* = 0.367, or main effect for Group, *p* = 0.517, was found. A significant main effect for Condition was found, F(1,36) = 16.880, *p* ˂ 0.001, η^2^ = 0.319. Post hoc comparisons revealed that, for the concussed group, a higher accuracy was obtained post-exercise (95.2%) compared to the dual task alone (93.1%), t(17) = −2.284, *p* = 0.036, d = 0.04. Similar results were found for the control group (dual-task ACC = 92.3%; post-exercise ACC = 95.5%), t(19) = −3.539, *p* = 0.002, d = 0.04. Thus, both groups demonstrated similar switch accuracy performance both pre- and post-exercise, and both groups exhibited better results post-exercise compared to the dual task alone (see Figure 2).

Figure 2 indicates that both groups demonstrated a similar switch accuracy between sessions. Furthermore, both groups exhibited a significant increase in accuracy after exercise (mean score by group/* significant differences).

#### 3.2.2. Switch Reaction Time

**Figure 3:**
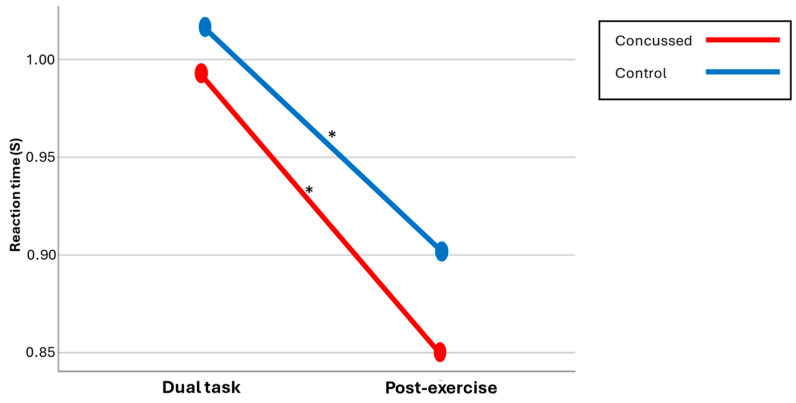
Cognitive reaction times across both conditions.

No significant Group × Condition interaction, *p* = 0.535, or main effect for Group, *p* = 0.253, was found. A significant main effect for Condition was observed, F(1,36) = 36.242, *p* < 0.001, η^2^ = 0.502. Post hoc comparisons revealed that, for the concussion group, a shorter average reaction time was obtained post-exercise (850 ms) compared to the dual task alone (991 ms), t(17) = 3.892, *p* = 0.001, d = 0.15. A similar result was observed for the control group (dual-task RT = 1012 ms; post-exercise RT = 902 ms), t(19) = 4.840, *p* < 0.001, d = 0.11. Thus, both groups demonstrated similar average switch reaction times during each session, and both groups exhibited improved post-exercise results compared to the dual task alone (see Figure 3).

Figure 3 illustrates that both groups significantly decreased their reaction time after the exercise, indicating an improvement in performance. There was no significant group difference (mean score by group/* significant differences).

### 3.3. Gait Outcome

**Figure 4:**
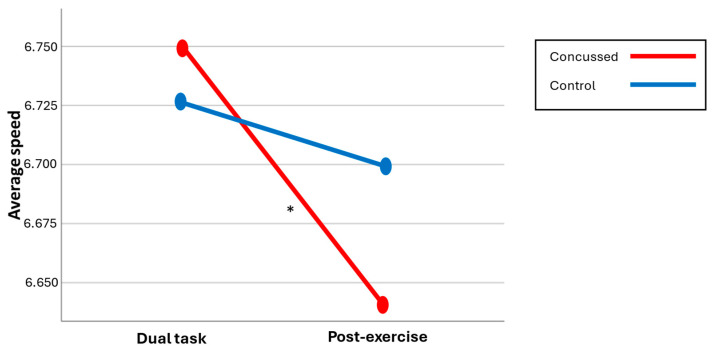
Gait speed across both conditions.

A significant Group × Condition interaction was found, F(1,36) = 4.42, *p* = 0.042, η^2^ = 0.11. Post hoc comparisons revealed that, for the concussion group, the average gait speed was significantly slower post-exercise (6.64 ± 0.14 km/h) compared to the dual task alone (6.75 ± 0.13 km/h), t(17) = 3.006, *p* = 0.008, d = 0.15. This pattern was not observed in the control group, *p* = 0.153. No difference was found between groups under the dual-task condition (*p* = 0.543) or the post-exercise condition (*p* = 0.113). Hence, even if both groups demonstrated similar performances in each condition, the concussion group’s pace was significantly reduced after the exercise condition compared to the no-exercise condition (see Figure 4). The control group performed similarly in both conditions.

Figure 4 illustrates a significant interaction that indicates a difference only present within the concussion group; these athletes tend to significantly reduce their average speed post-exercise (mean score by group/* significant differences).

## 4. Discussion

The present study aimed to investigate the effect of a single bout of moderate aerobic exercise on complex dual-task performance in athletes with a history of concussion compared to athletes with no history of concussion. Overall, our findings indicate that engaging in a moderate bout of aerobic exercise enhances the cognitive performance of all athletes. However, athletes with a history of concussion experienced a significant reduction in gait speed when completing the dual task after exercise, while no change was observed for the control group.

The omnibus analysis investigating switch task performance did not reach significance. However, the exploratory post hoc analyses revealed that both groups were more accurate and had faster reaction times after the exercise protocol. There are two possible interpretations of the improvement in cognitive performance observed after the exercise protocol. First, exercise enhances cognitive functioning, which aligns with extensive research indicating that a single exercise session increases cognition in healthy individuals [27,28,29,30]. The second possible explanation is a practice effect, as the conditions were not counterbalanced. In this study, to minimize potential carry-over effects, participants first completed the dual-task condition without prior physical exertion and then the combined post-exercise and dual-task condition. This sequence might have resulted in improved switch task performance in the second session due to a practice effect. However, this was mitigated by using alternate versions of the switch task. Previous work from our group found that the practice effects caused by repeated testing with the switch task are reduced, though not completely eliminated, by the use of alternate versions of the task [26]. One concern is that a practice effect, due to the lack of counterbalancing, could mask potential exercise-induced cognitive deficits in concussed athletes. However, if such cognitive deficits were present, we would expect them to at least partially negate the practice effect. If the practice effect is masking deficits, these deficits are likely subtle and potentially not of significant clinical consequence.

Gait speed significantly decreased following the single bout of moderate aerobic exercise in the concussion group, while no such change occurred in the control group. It is possible that concussed athletes slowed their gait to maintain cognitive performance. This aligns with studies indicating that, during dual tasks, more attentional resources are allocated to the more challenging task [36,37]. Additionally, the learning progress motivation hypothesis suggests that engaging in challenging tasks enhances liking, engagement, and performance compared to easier tasks [38]. Therefore, due to the negative effect of concussion on cognition, it is plausible that participants prioritized the cognitive task, dedicating more effort and attention to it at the expense of the gait task. This is consistent with a growing body of research showing altered cognitive functioning following concussion [39,40]. The present findings are consistent with most concussion studies using dual-task paradigms, which often report an increase in gait dual-task cost, though not always in cognitive dual-task cost [23,41,42]. An alternative explanation is that concussion could affect neuromuscular responses or enhance muscular fatigue. However, it is worth noting that all athletes in the concussion group had fully returned to play, making it unlikely that they were affected by physical deconditioning. Additionally, to our knowledge there is no substantial evidence in the scientific literature to support this hypothesis. Previous studies, including research from our lab using similar dual-task paradigms, have shown that concussion significantly affects gait when performed as part of a dual task but not when performed alone [22,23,43,44].

Interestingly, in our study, both groups tended to exceed the target speed in the no-exercise and post-exercise conditions. It is possible that their propensity to go faster is due to the overtaxing of their attentional resources. This finding provides valuable insight into how athletes respond to our protocol.

Our results indicate that the combination of a dual task with a post-exercise protocol reveals persistent gait alterations in individuals who are asymptomatic and had fully returned to their sporting and daily activities. The current findings could explain, at least in part, the increased risk of musculoskeletal injuries associated with concussion [45]. For example, a previous study revealed a relationship between worse gait dual-task cost and the risk of sustaining subsequent time-loss injuries [45]. In that study, participants were tested at two time points: (1) within the first 21 days post-concussion and (2) when clinically cleared to return to play. They also completed a questionnaire one year later. The participants performed a dual task that required them to walk at a self-paced speed in a straight corridor while simultaneously engaging in a working memory task (spelling a five-letter word backwards). The results showed that athletes who experienced worsening dual-task cost (i.e., slower average gait speed) were more likely to sustain a subsequent time-loss injury. Although this study bears many similarities to ours, our protocol included more complex gait and cognitive tasks, as well as physical exertion.

The clinical implications of our protocol align with current consensus recommendations [19] advocating for dual-task assessments. Our proposed dual task closely mirrors the dynamic nature of sports activities, offering a more ecologically valid approach that may better capture the challenges faced by athletes recovering from concussion. Incorporating a physical exercise component further enhances the sensitivity of our protocol, potentially uncovering subtle performance changes that conventional evaluations might miss. Our findings suggest that this integrated approach can effectively detect impairments in asymptomatic athletes who resumed play in the long term. Examining its impact on symptomatic athletes could provide valuable insights into its clinical relevance. Implementing this protocol may enhance the safety of post-concussion management by aiding decisions on return-to-play protocols.

### Limitations

First, one potential limitation of the present study is the requirement for all participants maintain a fixed speed of 6.5 km/h during the gait task. This differs from most studies using dual tasks, during which participants are required to walk at a self-selected pace. Hence, our approach could pose a potential problem, as a fixed speed could present a varying challenge for people of different heights [46]. However, this cannot explain our overall pattern of results, given that the average height of participants did not differ significantly between the control and concussed groups (mean: Control = 177.25 cm, Concussion = 177.28; *p* = 0.992). Second, the participants were not matched for their level of education. Some variables that normally correlate with this, such as IQ, could have had an impact on our results [47]. However, supplementary control analyses that compared the different outcome measures with the level of education revealed no significant differences (gait, switch accuracy, and reaction time ps > 0.407). Third, our results can only be generalized to the post-acute phase of recovery, as the athletes who took part in the study experienced their last concussion between 3 and 10 months before testing and had recovered and returned to normal activities. Prospective studies are needed to better understand how athletes in the acute phase of their injury, or those still experiencing symptoms, differ from control athletes in the dual task performed following exercise. Finally, the lack of counterbalancing, chosen to minimize potential carry-over effects, introduces the possibility of a practice effect masking exercise-induced cognitive deficits in concussed athletes. While we mitigated this concern by using alternate versions of the switch task, some residual practice effect might still be present. However, if this practice effect is masking deficits, they are likely subtle and potentially not of significant clinical consequence. Despite this, it is unlikely to have impacted the overall pattern of results, which demonstrated that asymptomatic concussed athletes experienced a significant reduction in speed while performing a dual task following a single bout of moderate aerobic exercise.

## 5. Conclusions

Using a post-exercise dual-task protocol akin to conditions athletes may encounter in their sport practice reveals gait alterations in asymptomatic concussed athletes long after their injury. These results highlight the added value of a dual-task condition combined with an exercise protocol in assessing recovery following concussion. The results presented in this study also highlight the importance of using a dual task in clinical environments. This paradigm has already been presented as a more sensitive approach to concussion assessment [22] and is recommended by the Concussion In Sport Group (CISG) [19]. Furthermore, our results, along with previous research [7,16,17,18], strongly support the added value of combining dual-task assessments with moderate aerobic exercise to evaluate post-concussion recovery.

## Figures and Tables

**Table 1:** Demographic information.

	Concussion (*n* = 18)	Control (*n* = 20)
Age in years, Mean ± SD	20.91 ± 2.63	19.95 ± 1.92
Sex, *n* (%)		
Male, *n* (%)	14 (77.8%)	13 (65.0%)
Female, *n* (%)	4 (22.2%)	7 (35.0%)
Years of education, Mean ± SD	15.00 ± 2.12	13.85 ± 2.03
Sports, *n* (%)		
Rugby	10 (55.6%)	4 (20.0%)
Hockey	2 (11.1%)	0 (0.0%)
Football	2 (11.1%)	2 (10.0%)
Soccer	1 (5.6%)	3 (15.0%)
Volleyball	1 (5.6%)	4 (20.0%)
Swimming	0 (0.0%	1 (5.0%)
Basketball	1 (5.6%)	4 (20.0%)
Cheerleading	1 (5.6%)	2 (10.0%)
Beck Anxiety Inventory, Mean ± SD	3.78 ± 4.49	4.90 ± 3.95
Beck Depression Inventory-II, Mean ± SD	6.00 ± 5.25	4.95 ± 4.56
Wender-Utah Rating scale, Mean ± SD	17.05 ± 15.02	13.05 ± 8.92
Time between sessions (days) Mean ± SD	10.06 ± 9.46	10.40 ± 8.82

## Data Availability

The data presented in this study are available on request from the corresponding author due to ethical reasons.
